# Comparative Transcriptome Profiling of Human Foreskin Fibroblasts Infected with the Sylvio and Y Strains of *Trypanosoma cruzi*

**DOI:** 10.1371/journal.pone.0159197

**Published:** 2016-08-09

**Authors:** Genevieve A. Houston-Ludlam, A. Trey Belew, Najib M. El-Sayed

**Affiliations:** 1 Department of Cell Biology and Molecular Genetics, College of Computational, Mathematical and Natural Science, University of Maryland, College Park, Maryland, United States of America; 2 Center for Bioinformatics and Computational Biology, University of Maryland Institute for Advanced Computer Studies (UMIACS), University of Maryland, College Park, Maryland, United States of America; Universidad de Chile, CHILE

## Abstract

*Trypanosoma cruzi*, the causative agent of Chagas Disease, is phylogeneticaly distributed into nearly identical genetic strains which show divergent clinical presentations including differences in rates of cardiomyopathy in humans, different vector species and transmission cycles, differential congenital transmission in a mouse model, and differing immune and heart inflammation response in dogs. The population structure of these strains divides into two groups, which are geographically and clinically distinct. The aim of this study was to compare the transcriptome of two strains of *T*. *cruzi*, Sylvio vs. Y, to identify differences in expression that could account for clinical and biochemical differences. We collected and sequenced RNA from *T*. *cruzi*-infected and control Human Foreskin Fibroblasts at three timepoints. Differential expression analysis identified gene expression different timepoints in Sylvio infections, and between Sylvio and Y infections in both parasite and host. The Sylvio strain parasite and the host response to Sylvio infection largely mirrored the host-pathogen interaction seen in our previous Y strain work. IL-8 was more highly expressed in Sylvio-infected HFFs than in Y-infected HFFs.

## Introduction

*Trypanosoma cruzi*, a protozoan parasite, is the causative agent of Chagas Disease, a disease which affects 6–7 million people primarily in Latin America. [1] The parasite is transmitted by a triatomine insect vector, where it is introduced into the host by the feces of the insect as it takes a blood meal. There is no preventive vaccine, and the current drug regimens are highly toxic. The drugs are fairly effective if taken during the early (acute) stage of the disease, with limited utility in the chronic stages of the disease. Approximately 30% of infected individuals will develop chronic Chagas Disease, which can manifest as inflammatory heart disease, and/or gastrointestinal disease leading to megacolon or megaesophagus. [2]

*T*. *cruzi* reproduces clonally, but there appears to be at least one historical hybridization event leading to strains with different haplotypes. The population structure is described by assigning the various strains of *T*. *cruzi* into six discrete typing units (DTUs) based on molecular markers. [3] While there is some debate as to the number of hybridization events that have occurred to produce the heterozygous strains in DTUs V and VI, there is broad agreement that DTUs I and II are pure ancient lineages. [4]

DTU I is the dominant strain of *T*. *cruzi* in Northern South America and Central America. It is believed that DTU I parasites differ from DTU II in many aspects. Those include clinical symptoms (such as the relative increase in gastrointestinal symptoms associated with DTU II parasites [5] and altered ECG [6]) in humans, differential congenital transmission in a mouse model [7], differing immune and heart inflammation response in dogs [8]. Robust epidemiological studies tying disease symptoms to parasite strain are lacking, although a recent study suggested that the rate of Chagas cardiomyopathy may be lower in countries with DTU I as the dominant strain compared to DTU II dominant countries [1]. In addition, different vector species and transmission cycles have been documented for DTU I and DTU II [9].

In this study, we aimed to investigate whether the Sylvio (DTU I) and Y (DTU II) strains of *T*. *cruzi* showed global transcriptome differences at the parasite and host cell level given the different clinical manifestations found.

## Materials and Methods

### HFF culture

Low-passage primary human foreskin fibroblasts (HFF) BJ strain (ATCC CRL-2522) were propagated in Dulbecco's Modified Eagle Medium (DMEM, Gibco) with 10% fetal bovine serum (FBS, Gemini Bioproducts), 1x MEM Non-essential Amino Acids (Cellgro) and 100 units Penicillin/100 μg Streptomycin/0.25 μg Amphotericin per mL (DMEM-10% FBS) and incubated at 37°C in 5% CO_2_. Following expansion, 2.5x10^5^ cells were seeded into 6-well plates in DMEM-10% FBS for 48 hours. Identical wells were prepared for microscopy with glass microscope coverslips for parasite burden computation. Two biological replicates, each originating from separate freeze dates after expansion from the original ATCC culture, were performed.

### Parasite infection

*Trypanosoma cruzi* strains Sylvio (ATCC 50800) and Y (obtained from the laboratory of Barbara Burleigh at Harvard University) were propagated in monolayers of Vero cells in DMEM (Cellgro) with 2% heat-inactivated FBS (Gemini Bioproducts), 1x MEM Non-Essential Amino Acids (Cellgro) and 100 units Penicillin/100 μg Streptomycin per mL (DMEM-2% FBS). Tissue Culture Trypomastigotes (TCTs) were harvested from the supernatant, centrifuged and resuspended in DMEM-2% FBS. Parasites (3.0x10^7^ parasites per well, 100:1 infection MOI) were incubated with HFF cells for 2 hours at 37°C at 5% CO_2_ to allow invasion. The remaining extracellular parasites were aspirated, each well was washed 5 times with PBS, fresh DMEM-2% FBS medium was added, and cultures were incubated for the indicated times. Timepoints were taken at 4, 12, 20, 24, 30, 48, 72 and 96 hours post-infection (hpi). Each biological replicate originated from a different stabilate of Sylvio or Y strain after expansion from the original culture.

### RNA sequencing

Media was aspirated from wells and 1 mL Trizol^®^ reagent (Life Technologies) was applied to the cell culture. The Trizol mixture was then collected and immediately placed in a -80°C freezer until total RNA could be isolated according to the Trizol protocol. Residual DNA was degraded using DNase and the remaining RNA was purified with a Qiagen RNeasy mini kit. RNA quantification and quality were assessed with an Agilent 2100 Bioanalyzer. PolyA+ cDNA unstranded libraries were prepared with the Illumina TruSeq sample preparation kit V2 (San Diego, CA, USA) using the Illumina Indexes for multiple samples per lane. Libraries were checked for quality and quantity using the Agilent Bioanalyzer 2100 and qPCR (KAPA Biosystems), then sequenced on an Illumina Hi-Seq 1500.

### Parasite burden computation

Wells with coverslips were fixed with 4% paraformaldehyde for ten minutes. The coverslips were washed five times with PBS, mounted onto microscope slides with ProLong Gold antifade reagent along with DAPI stain (Life Technologies) and allowed to set overnight at room temperature in the dark. Parasites were counted at 100x oil immersion on a Zeiss AxioObserver Microscope. One hundred cells on each coverslip were counted, with three coverslips per timepoint. Percent infected HFF cells and parasite burden per infected cell were computed.

### RNA-Seq data processing and mapping

Paired-end reads (100 bp) were demultiplexed then trimmed for residual adapter sequences using Trimmomatic (Version 0.32) [10], with the following parameters: paired-end reads, phred33, ILLUMINACLIP:/TruSeq3-PE.fa:2:30:10, LEADING:20, TRAILING:20, MINLEN:36. Trimmed reads were checked for per-base quality using FastQC (Version 0.11.2) [11]. Reads were aligned with Tophat [12] (version 2.0.10, which invoked Bowtie version 2.1.0.0 and Samtools version 0.1.19.0) using default parameters except–r 170 to specify mean inner distance between mate pairs. Tophat was provided the appropriate reference genome for the species being analyzed (hg19 for human, TriTrypDB version 4.1 for CL Brener Esmeraldo-like haplotype (proxy genome for Y-strain parasite) [13] or TriTrypDB version 6.0 for Sylvio parasite [14]. Two mismatches per read were allowed and reads were allowed to map only to a single locus. The number of reads mapping to each gene feature in the corresponding annotation files was determined using HTSeq [15].

### Sample assessment by statistical testing and visualization

Data for human and parasite samples collected in this study and data from our previous work were checked for consistency between replicates using a variety of global inter-sample correlation analyses including Pearson correlation, median pairwise correlation (MPC), box plots, principal component analysis (PCA) and Euclidean distance-based hierarchical clustering on quantile normalized, log_2_ transformed data. A standard method for identifying outliers was applied to the data samples [16]. The median pairwise correlation (MPC) for the first quartile across samples was computed (Q1), as was the inter-quartile range (IQR). Briefly, any sample whose MPC was less than Q1-1.5(IQR) was removed as an outlier. Two samples were removed from the human data based on this criterion. No parasite samples were removed.

### Differential expression analysis

Weakly expressed genes, defined as having less than 1 read per million in ‘n’ of the samples, where ‘n’ is the size of the smallest group of replicates [17] (here n = 10 and 6 for the *T*. *cruzi* and human samples, respectively) were removed from subsequent analyses. Data were quantile normalized and log_2_ transformed [18]. The list of differentially expressed (DE) genes was determined using voom [19] to account for the mean-variance relationship, then the data were fitted to a linear model, which included batch to account for experimental batch effects in the data, using limma [20]. Storey’s q-value was applied at an FDR cutoff <0.05 to determine significance [21].

### Parasite Ortholog Identification

Version 8.0 genome fasta files for *T*. *cruzi* Sylvio strain and *T*. *cruzi* CL Brener strain (Esmeraldo-like haplotype) were evaluated to identify uniquely mapping orthologs. The FASTA program ggsearch36 [22], which does a global:global alignment, was used with default parameters (E-value < = 0.001, score > 0). Reciprocal best hits were then further refined by removing genes in the four largest multi-gene families (MASP, trans-sialidase, retroposon hot-spot and dispersed gene family protein), thereby avoiding the problems introduced by assembly of highly similar and repetitive genes. Altogether there were 4,659 unique orthologs, of which 3,279 (70%) were annotated as hypothetical proteins. This set of genes is referred to as the “core” set of parasite genes.

### Gene Ontology Analysis

The GO Term Finder [23] was applied to the human and parasite data using the server at go.princeton.edu. For the human data, Ensembl IDs were converted to HUGO Gene Nomenclature Committee Database (HGNC) IDs with the BioMart ID Conversion tool, then GO Term Finder was applied with the “GOA + HGNC Xrefs- H. sapiens (human)” annotation. The human data was also interrogated with g:Profiler [24] to simultaneously identify GO, KEGG and Reactome term enrichment. For *T*. *cruzi*, the GO terms were extracted from the Version 9.0 TriTrypDB text file into a standard gene association file (GAF format) and provided in the advanced input options.

### Access to sequence data and data analysis pipeline

Sequence data are available at the NCBI sequence read archive (SRA) under Bioprojects PRJNA251582 and PRJNA251583. Accession numbers for each individual sample are provided in S1 Table. All components of the data quality assessment statistical pipeline, named cbcbSEQ, were carried out in R and can be accessed on GitHub [25].

## Results and Discussion

### Experimental Design

We investigated whether we could detect differences in the global transcriptomes of two *T*. *cruzi* strains: Sylvio (DTU I) and Y (DTU II), as well as host cells given the distinct clinical and biochemical profiles suggested by previous reports. Using RNA-Seq, we simultaneously interrogated the transcriptomes of the host and parasite over the course of intracellular infection. Two independent Sylvio infections were carried out in HFF cells and RNA was harvested from pre-infection tissue culture trypomastigotes (TCT) as well as infected cells; these were matched with uninfected control cells at 4, 24 and 72 hpi. Although the bulk of the Y strain data used was derived from our previous work [26], we performed a similar experiment with Y strain-infected HFF cells to check for consistency across experimental dates and to allow us to evaluate batch effects.

Total RNA from human and parasite was isolated for each of the two biological replicates and Poly(A)+ enriched cDNA libraries were generated and sequenced using the Illumina HiSeq 1500 platform. A total of ~1.1 billion reads were produced across 16 samples, with a mean of 94% mapping to either the host or pathogen reference genomes (S1 Table). Reads were counted on a per-gene basis using the reference annotations for the host and pathogen, producing count tables for statistical and differential expression (DE) analysis.

**Fig 1:**
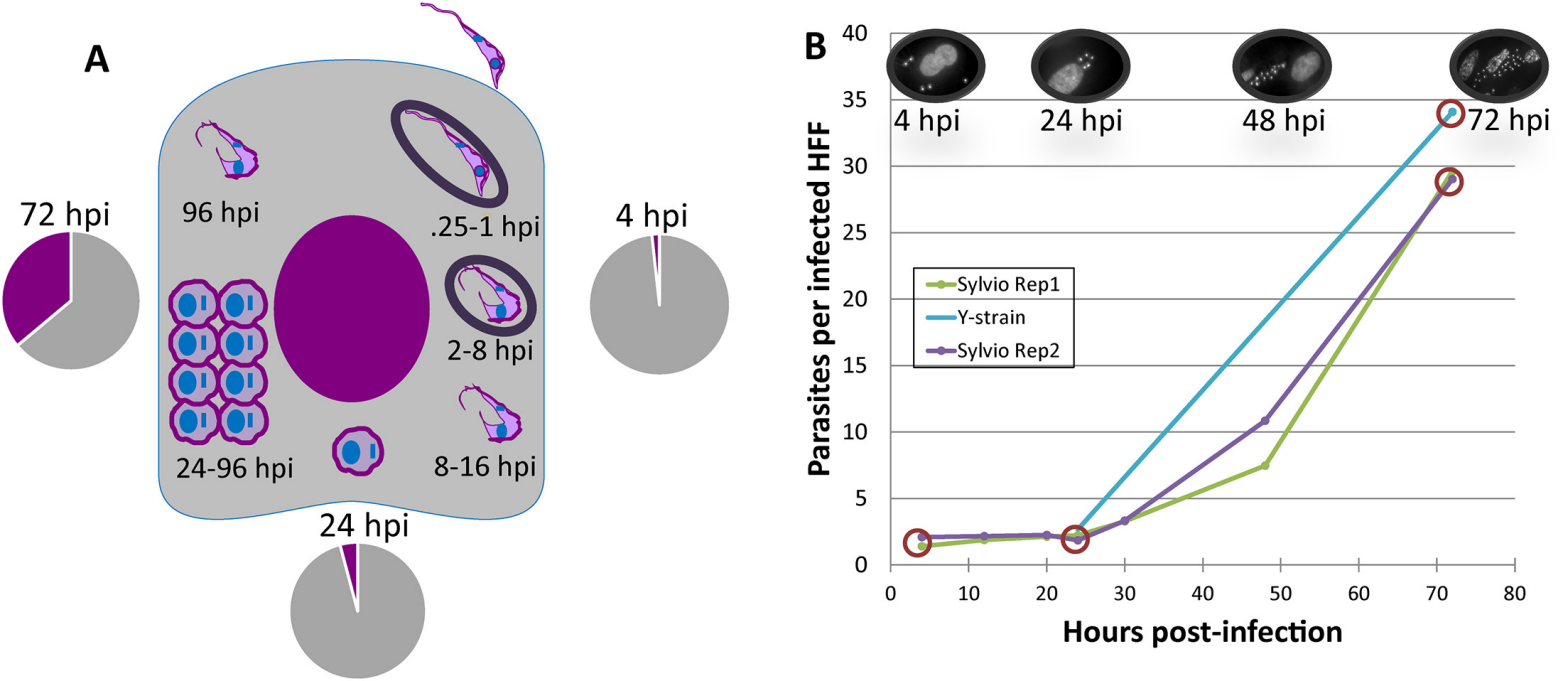
Study design and infection dynamics of two strains of *Trypanosoma cruzi*. **A.** Schematic representation of samples collected from the infection of human foreskin fibroblasts (HFF) with *T*. *cruzi* Sylvio strain. The expected progression of the *T*. *cruzi* infection is shown. Briefly, the parasite invades the cell within 15 minutes of attachment, and is enclosed by a portion of the host cell membrane (parasitophorous vacuole). By 4 hours post-infection (hpi), the parasite begins to transform from infective trypomastigote to the intracellular amastigote; it exits from the parasitophorous vacuole at around 8 hpi. The parasite completes its transformation by 24 hrs and starts dividing. By around 96 hpi, the parasite begins the transformation back to the trypomastigote form. The trypomastigotes then escape the cell and the infection process begins anew. Two independent infections were carried and total RNA was collected at three time points (4, 24 and 72 hpi), from both infected cells and tissue culture typomastigotes. Libraries were prepared and sequenced for each of the two biological replicates at each timepoint. Pie charts show the average proportion of RNAseq reads mapping to the human (gray) and parasite (purple) reference genomes for each of the intracellular timepoints. Mapping details are provided in S1 Table. **B**. Parasite burden per cell is shown for each timepoint in each experiment. One hundred DAPI-stained cells were counted on each of three independent coverslips on a Zeiss AxioObserver microscope at 100x oil-immersion. A parallel Y-strain infection was run for a comparison with the existing Y strain dataset [26]. Samples from circled timepoints were sequenced.

The results of the infection experiments are summarized in Fig 1. The replicate infections with *T*. *cruzi* Sylvio strain behaved consistently with an approximate parasite burden of two parasites per infected cell at 4 hpi and about 65% of the host cells infected. Parasite division was observed beginning at 24 hpi. Both Sylvio and Y strains displayed similar infection dynamics (Fig 1B) and behaved consistently with the existing Y strain dataset [26]. Most importantly, both Sylvio and Y strains followed the expected time course projection of an experimental *T*. *cruzi* infection as previously described [27].

Because RNA-seq reads were mapped unambiguously to the host and parasite reference genomes, we were able to track the respective fractions of RNA for each species in each sample. Our analysis revealed a proportion of 1.7% of the total mRNA population attributable to the parasite early in the infection (4 hpi) (Fig 1A). The parasite’s contribution increased to 4.2% at 24 hpi, consistent with the expected doubling beginning at around that point in the infection. By 72 hpi, the parasite RNA proportion increased to 36.1%.

### Global statistical evaluation of samples used for comparative transcriptome analyses

We used various statistical methods to evaluate the consistency between biological replicates and examine global trends in underlying distributions of per-gene counts within and between parasite strains. In order to identify outliers, we carried out a median pairwise correlation analysis for each sample against all other samples within each species. Two human samples fell below the 1.5 times inter-quartile range cutoff, and were removed from further consideration (S1A Fig). The parasite data showed good correlation between biological replicates within each strain (S1B Fig).

**Fig 2:**
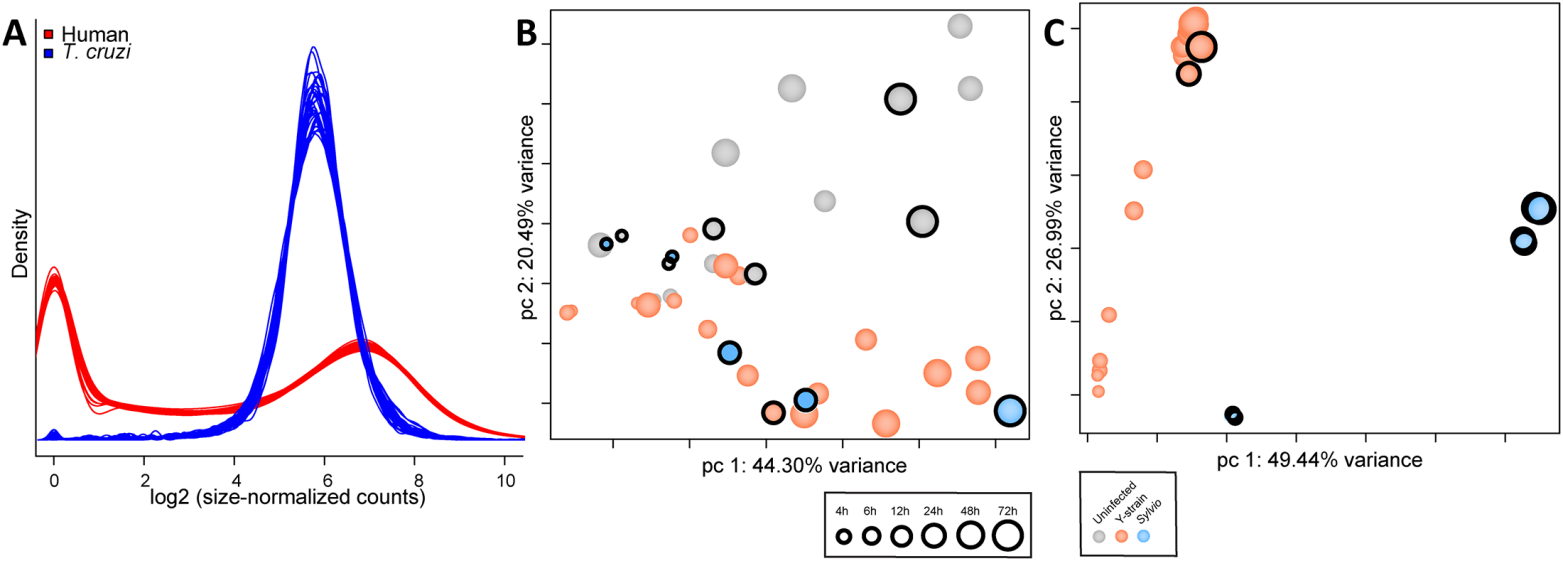
Combined statistical evaluation of all samples used in comparative transcriptome analysis. **A.** Density Plot showing the estimated underlying distribution of abundance of reads mapping to each gene feature in the corresponding annotation files. Data is shown for all human (red) and parasite (blue) samples from this study (Sylvio and Y) as well as the Y strain dataset from our previous study [26]. Principal Component Analysis (PCA) of all human (**B**) and parasite (**C**) samples following low count genes removal, quantile normalization, and accounting for batch effects in the statistical model. Parasite data was limited to the set of 4,659 single-copy core genes shared between Sylvio and Y strains. Time (hpi) is represented by increasing size of circle. Infection status and strain is depicted by color of circles. Circles with a black ring identify datapoints produced in this study.

A density analysis showing the estimated underlying distribution of abundance of reads mapping to each gene feature showed that the distribution of counts was consistent within human and *T*. *cruzi* samples (Fig 2A). Interestingly, this density plot highlighted different aspects of gene expression regulation in human and parasite genes. Human gene expression is frequently regulated at the transcriptional initiation level, with many genes turned off under different conditions [28], and this is reflected in the spike at zero count. The *T*. *cruzi* parasite (like other trypanasomatids) transcribes the majority of its genes in polycistronic units [29] and fine-tunes steady-state mRNA levels post-transcriptionally. A narrow range of mRNA abundances is observed with a single peak distribution.

A principal component analysis was performed on normalized per-gene counts from both species, incorporating data produced in this study with the Y strain data from our previous study [26]. This allowed us to evaluate any potential batch effects introduced by the different isolations and the different experimental timeframes. For the human data (Fig 2B), principal component 1 (PC1) showed a progression of time post-infection, with early timepoints clustering on one end and later timepoints on the other. Control (uninfected) samples clearly separated from infected samples along PC2, with the distance between control and infected cells growing larger as the infection progressed. Notably, data from this study, highlighted with black circles, grouped tightly with the data produced previously despite the experiments being run in two different collaborating laboratories more than a year apart.

The parasite data (Fig 2C) showed a clear separation along PC1 between *T*. *cruzi* Sylvio and Y strains; the 4 hour Sylvio timepoint clustered close to the corresponding Y strain data and the distance increased as the infection progressed. PC2 reflected the progression of time post-infection. The early timepoints (4–6 hpi) clustered tightly, when the parasite is still expected to be in the parasitophorous vacuole and is beginning transition from the infective trypomastigote into the intracellular replicative form. The datapoints from 12 hpi, when the parasite would have left the parasitophorous vacuole, clustered between the early and late timepoints. Finally, the data from the later timepoints, 24 to 72 hpi, when the parasite would be dividing among other things, clustered together tightly. The Y strain datapoints produced in this study grouped with the analogous Y strain timepoints from /our previous study.

The separation of Y and Sylvio strain was expected considering that the parasite PCA analysis was limited to a core orthologous gene set defined in our study. This reduced dataset excluded many multi-gene family members which include many of the virulence factors associated with attachment and invasion and have generally been presumed to drive biological differences between strains [30].

### Differential expression analysis of the host cell response to two strains of *T*. *cruzi*

**Fig 3:**
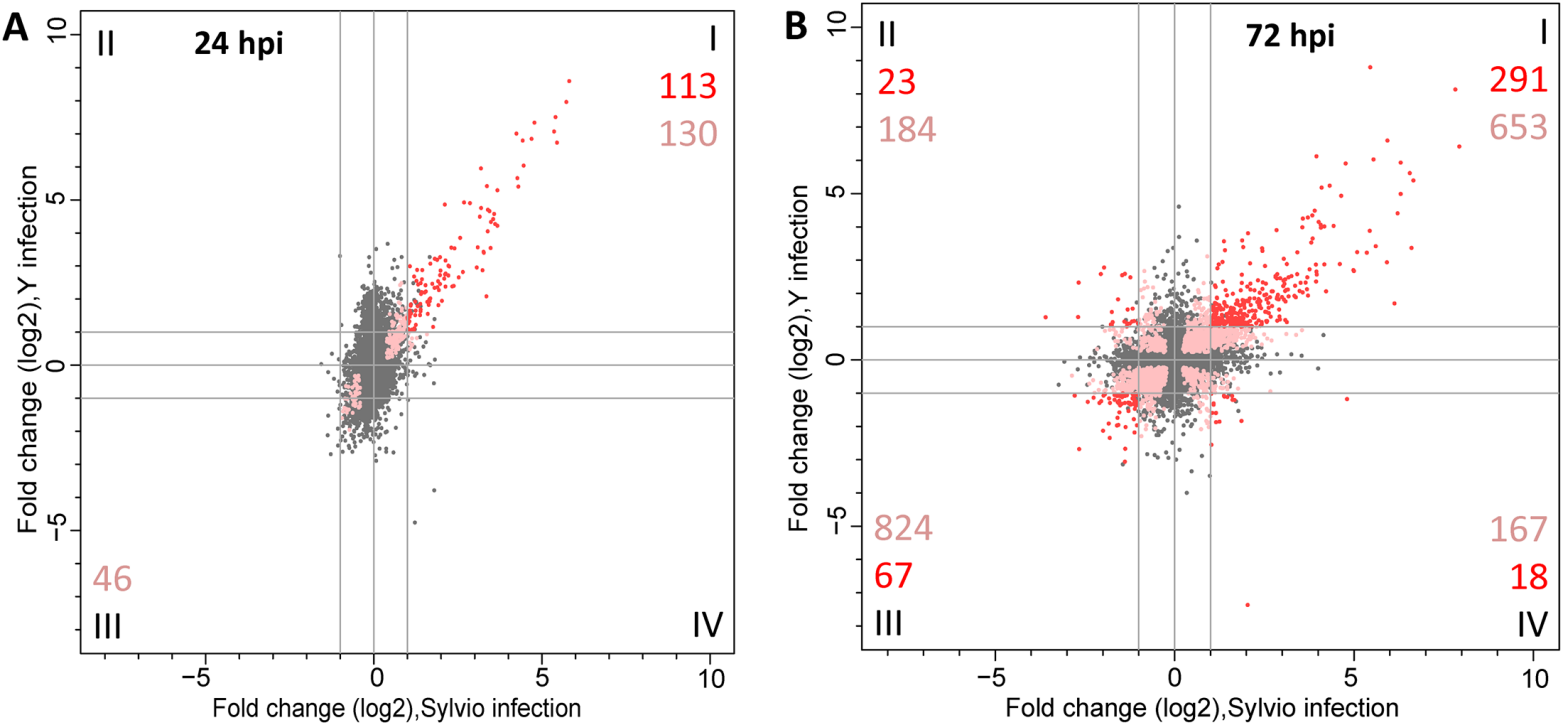
Comparative analysis of the host transcriptome response to infection with Sylvio versus Y strain. Scatterplot of log_2_ fold change in expression of human genes in HFF cells infected with Sylvio (x-axis) versus Y (y-axis) at 24 hpi (**A**) and 72 hpi (**B**). Genes that were not significantly differentially expressed are shown as gray dots. Pink dots represent significantly DE genes in infections with both strains but had log_2_ FC in one or both strains that was below our cutoff value of 1. Red dots depict genes that were significantly DE and had absolute value of log_2_ FC>1 in both strains. The numbers in each quadrant correspond to the total number of significantly DE genes that met the log2 FC cutoff of 1 (red) or were significantly DE but did not meet the FC cutoff (pink).

A comparative differential expression analysis was performed to interrogate the response of human host cells infected with the two different strains of *T*. *cruzi*. We generated DE profiles for HFF cells infected with *T*. *cruzi* Sylvio and compared them to the DE lists generated for our *T*. *cruzi* Y infection in our previous study [26]. A graphical representation of the similarities and differences in the host response to infection with the two different strains of the *T*. *cruzi* parasite is shown in the form of a scatter plot to visualize the shared expression signature (Fig 3). While we did not identify significantly differentially expressed genes in *T*. *cruzi* Sylvio-infected cell at 4 hpi (in contrast with 441 DE genes at the same time point in Y strain-infected cells), we observed a strong similarity between the host response to Sylvio and Y strains of *T*. *cruzi* at 24 hpi and later when genes that are significantly DE in both infections (and had a fold change greater than 2) are compared. While this observation may suggest that a Sylvio infection is more silent in the early hours of a mammalian infection, more biological replicates would be needed to make such an assertion. At 24 hpi, HFFs infected with Sylvio or Y shared a common set of 113 genes that were significantly upregulated (Fig 3A, quadrant I). There were no significantly DE genes co-downregulated or upregulated exclusively in Sylvio or Y infections at 24 hpi. There was a marked increase in the number of shared genes significantly DE between 24 hpi and 72 hpi. By 72 hpi (Fig 3B), the number of genes that were significantly DE in HFFs infected with either Sylvio or Y in the same direction increased to 358. Of these, 291 (81%) were commonly upregulated and 67 (19%) were downregulated (Fig 3B, quadrants I and III respectively). An examination of these shared genes revealed that of the 113 genes significantly DE at 24 hpi, 102 (90.3%) were still significantly DE at 72 hpi. We conclude that most of the intermediate (24 hpi) response signature remained over the course of the infection with an additional modulation of host cell genes as the infection progressed.

Consistent with our previous work, the genes most upregulated in infections with both strains are associated with a type I IFN response. At 24 hpi, nine of the top ten genes (S3 Table) are induced by type I interferons, and are generally associated with antiviral activity. By 72 hpi there is a slight reordering of the top ten co-upregulated genes, with seven of the top ten at 24 hpi remaining in the top ten at 72 hpi, and the rest still within the top 15 DE genes at 72 hpi. The three genes which move into the top 10 at 72 hpi have pro-apoptosis and/or tumor suppressor activity. The gene with the largest increase between 24 and 72 hpi is tumor necrosis factor ligand superfamily member 10, also known as TNF-related apoptosis-inducing ligand (TRAIL, ENSG00000121858). This particular member of the TNF ligand superfamily is associated with inducing apoptosis in tumor cells, but not in normal cells [31].

A recent report showed a correlation between certain SNPs in the IL-18 gene and severity of chronic Chagas cardiomyopathy in a Columbian population [32]. A previous study in mice showed an increase in plasma IL-18 at day 6 in response to a *T*. *cruzi* infection [33]. In the Y strain, we found the gene for IL-18 (ENSG00000150782) was significantly upregulated at 24 hpi with a 2.4-fold change. At 72 hpi IL-18 was downregulated in Y strain 2.4-fold, but it did not meet the FDR cutoff of 0.05. The Sylvio strain showed a response more consistent with the mouse data, being upregulated 1.3-fold (not statistically significant) at 24hpi and significantly upregulated 3.3-fold at 72 hpi.

To evaluate the biological activity being performed by the host cell for this shared signature, we performed a Gene Ontology (GO) analysis as well as a KEGG analysis on the shared significantly DE genes. At 24 hpi, GO analysis of the co-upregulated genes revealed a general innate immune response, with Type I interferon response being the most vigorous. A similar enrichment was observed at 72 hpi. A KEGG analysis showed an upregulation in sterol biosynthesis pathway at 72 hpi.

There were no genes at 24 hpi that met the significance and fold change cutoffs in the co-downregulated genes, or the genes differentially regulated in the two different strains. At 72 hpi (S3 Table) the top two co-downregulated genes are involved in cell growth and development, and the third is a transcription factor mostly active in cardiac and smooth muscle gene regulation. A GO analysis of the significantly DE co-downregulated genes (Quadrant 3) showed a downregulation of the cell cycle. All of these observations were consistent with our previous work [26].

We infer from our observations that there are no general differences in the host cell transcriptomes when infected with either Sylvio or Y strain of *T*. *cruzi*, at least in this *in vitro* system and this specific cell line. Because these experiments were performed in cell culture, the factors associated with the global immune response in the context of different human genotypes in response to the parasite infection are not considered. It is worth noting that in Chagas disease, approximately 70% of infected individuals do not develop a chronic disease [34]. In these individuals, the infection is either resolved or contained.

There were a few detectable differences in the host cell response to the two strains which did not appear until 72 hpi. A total of 41 genes were upregulated in only one strain and downregulated in the other (S3 Table). Of those, 23 genes were upregulated in Y strain infections and downregulated in Sylvio infections (Fig 3B, quadrant II) and do not reflect any discernible signature. The top five genes consist of two transcription factors, a cell adhesion molecule, a protein associated with cognitive function and a protein associated with tissue enervation and CNS development.

The remaining 18 genes were upregulated in Sylvio infections and downregulated in Y strain infections (S3 Table, Fig 3B, quadrant IV). These consist of a ribosomal small subunit protein, two immune system cytokines, a metabolic enzyme that cleaves glutathione and a protein associated with meiosis. While we did not identify any GO categories that were significantly overrepresented among those genes, we note that the genes encoding IL-8 and IL-32 behave very differently in host cells infected by the two different *T*. *cruzi* strains. IL-8 is a chemokine which attracts monocytes to an area of injury or infection. IL-32 causes the production of pro-inflammatory cytokines and chemokines, including IL-8. One can speculate that the upregulation of these two cytokines in Sylvio infected cells could offer a partial explanation of the difference in the overall clinical picture between the infections with the two strains by attracting immune cells to respond to host cells infected with Sylvio. This biological relevance of this observation can only be ascertained by testing a greater number of replicates *in vitro* as well as establishing that similar expression patterns can be observed in patients infected with the two strains.

The details of the DE analyses for the human data are included in the supplementary materials. S2 Table contains the summary statistics for the gene counts described above. S3 Table contains the list of genes with their respective DE significance (qval) and fold change for both Y and Sylvio infections at 24 and 72 hpi as well as tabs for the genes displayed in red in Fig 3A and the GO analysis.

### Differential expression analysis of *T*. *cruzi* response during infection

We also examined the parasite transcriptome during the infection of a human host cell by generating DE profiles for *T*. *cruzi* Sylvio and comparing them to the DE lists generated for *T*. *cruzi* Y infection in our previous study [26]. All *T*. *cruzi* Sylvio-infected samples and the full set of genes in the Version 8.0 TriTrypDB Sylvio annotation were used for differential expression analysis. The analysis performed was identical to the one carried out on the human data described above, with some additional limitations inherent to the parasite genome(s). While the human genome is finished and well annotated, the parasite reference genomes used in this study are not fully assembled because of their significant repeat content and the multigene families. The CL Brener strain of *T*. *cruzi*, the original sequenced genome [13] represents the most ‘finished’ genome including chromosome-assigned scaffolds [35], whereas the Sylvio genome assembly consists of 19,000 contigs [36]. In addition, a large proportion (~60%) of the gene products in *T*. *cruzi* are annotated as ‘hypothetical’ and the gene ontology labels and KEGG pathway assignments are sparse.

We defined a set of 4659 uniquely matching orthologs (“core genes”) as described in the Materials and Methods (S6 Table). To generate this core set of orthologs, we selected unique reciprocal best hits for the two parasite strains and removed genes associated with multigene families. This allowed us to unambiguously compare expression levels for orthologous genes in both strains despite the different naming conventions for genes in Sylvio and Y strains.

**Fig 4:**
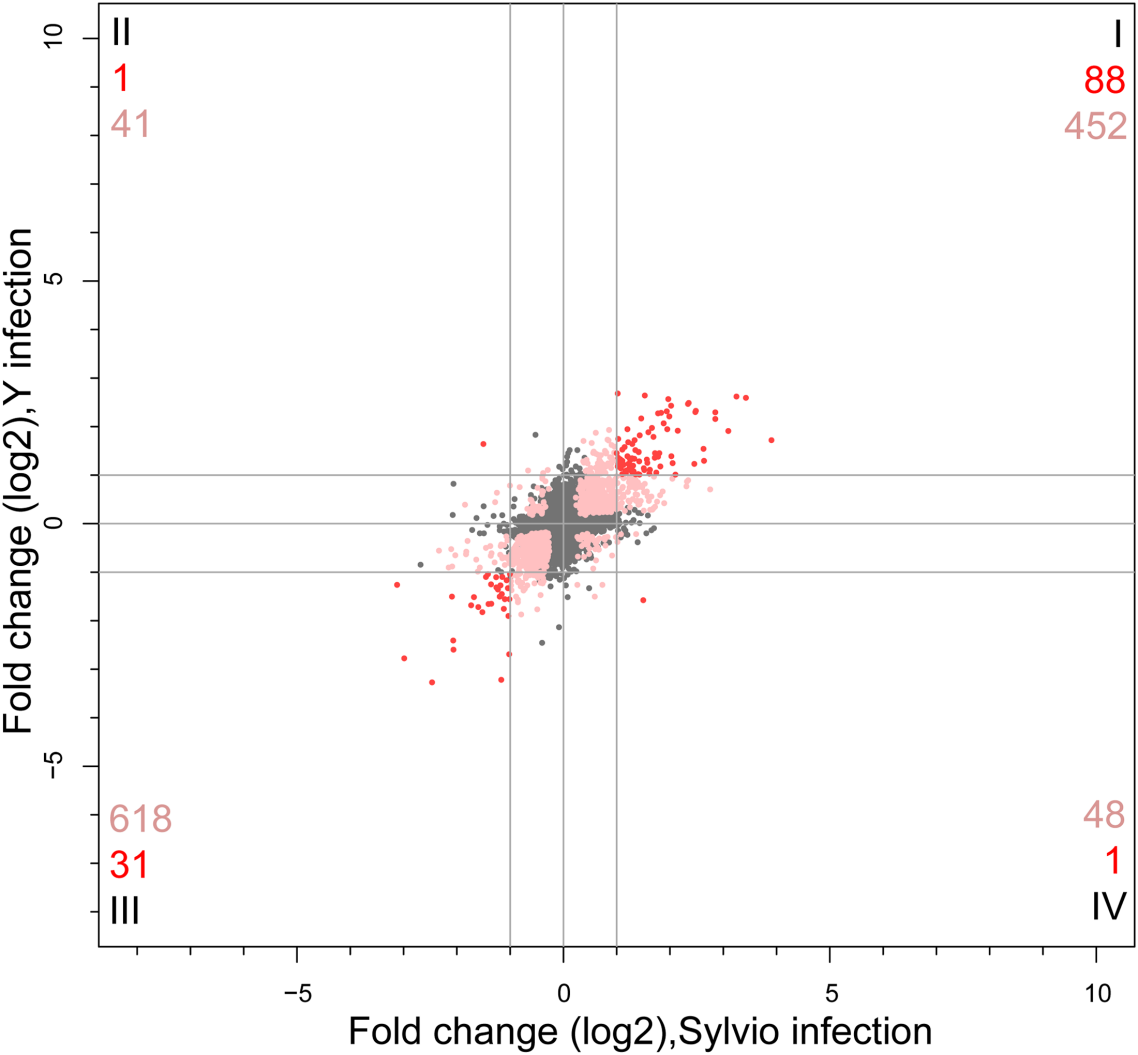
Comparative analysis of *T*. *cruzi* parasite Sylvio versus Y strain transcriptome response. Scatterplot of log_2_ fold change in expression of a subset of orthologous *T*. *cruzi* parasite genes in Sylvio (x-axis) versus Y strain (y-axis) at 24 hpi versus 4 hpi. Genes that were not significantly differentially expressed are shown as gray dots. Pink dots represent genes that were significantly DE in both strains of parasite but had log_2_ FC in one or both strains that was below our cutoff value of 1. Red dots depict genes that were significantly DE and had absolute value of log_2_ FC>1 in both strains. The numbers in each quadrant correspond to the total number of significantly DE genes that met the log_2_ FC cutoff of 1 (red) or were significantly DE but did not meet the FC cutoff (pink).

Similar to our observations in the host cell response to the two strains, a striking similarity was seen between the response of Sylvio and Y strains of *T*. *cruzi* when the significantly DE core genes were compared. A graphical representation of the similarities and differences between the two strains as the parasite transitions from the 4 to 24 hpi is shown in the form of a scatter plot (Fig 4). There was a common set of 119 core genes that were significantly DE at greater than 2 fold. Of these, 88 were commonly upregulated (Fig 4, quadrant I), and 31 were commonly downregulated (Fig 4, quadrant III) in both strains. Between 24 and 72 hpi, 4 shared genes were significantly DE shared genes at greater than 2 FC (S4 Table). The substantially larger number of genes differentially expressed between 4 and 24 hpi than between 24 and 72 hpi are consistent with our current understanding of the biology of the parasite during infection and our previous observations in *T*. *cruzi* Y strain. After invading the host cell, *T*. *cruzi* begins its transformation from the invasive trypomastigote form into the intracellular, replicative amastigote form. The parasite completes this transformation within the first 24 hpi, and during the next 72 hours the regulatory regime stays relatively static during division until it bursts the host cell sometime between 96 and 120 hpi.

The gene with the largest increase in co-expression between 4 and 24 hpi is glutamate dehydrogenase (TcCLB.507875.20/TCSYLVIO_003673). This gene is involved in the catabolism of glutamate, and is likely associated with the conversion of parasite metabolism from carbohydrates in the trypomastigote form to amino acids and fatty acids in the amastigote form [37,38]. The second highest mutually upregulated parasite gene is a chromosomal passenger protein, which localizes at the centromere and influences cell division. The third most co-upregulated gene is a pyruvate kinase containing gene, which is involved in glycolysis. Fourth is a hypothetical protein of unknown function, and fifth is methylthioadenosine phosphorylase, an enzyme which participates in the salvage of adenosine and methionine. We examined the shared parasite signature for Gene Ontology enrichment in the subset of genes significantly DE between 4 and 24 hpi. Analysis of all co-upregulated genes revealed an enrichment of lipid and sterol biosynthesis pathways, consistent with our previous observations in *T*. *cruzi* Y strain [26]. We also noted an enrichment of the one-carbon metabolism process (GO:0006730). This process produces the precursors for the production of trypanothione, a key metabolite responsible for addressing oxidative stress in trypanosomes, as trypanothione reductase mutants have been shown to be avirulent [39].

Of the 31 commonly downregulated genes, the two genes with the largest decrease in co-expression between 4 and 24 hpi are hypothetical proteins of unknown function. The third most co-downregulated gene is polyprenyl synthetase, involved in synthesis of cholesterol, ubiquinone and coenzyme Q10, all part of lipid metabolism. The fourth most co-downregulated gene is adenine phosphoribosyltransferase, which is part of the adenine salvage pathway, and the fifth is glutamine synthetase, which produces glutamine from glutamate and ammonia. The significantly DE co-downregulated genes showed no significant GO enrichment.

Only two significant DE genes meeting the fold change cutoff were unique to one strain or the other. One was a hypothetical protein (TcCLB.507143.60/TCSYLVIO_002455) upregulated ~3-fold in Y but downregulated ~2.8-fold in Sylvio. The other was an amino acid permease (TcCLB.511325.50/TCSYLVIO_010159) upregulated ~2.8-fold in Sylvio and downregulated ~3-fold in Y. S4 Table contains the summary of the results of the significantly differentially expressed analysis for the parasite data. A list of parasite genes meeting the significance and fold change filters are shown in S5 Table.

## Concluding Remarks

Our analysis did not elucidate substantial differences between either the host or the parasite response to infection in this *in vitro* system. The relatively small changes between parasites may be explained by constraints levied by the *in vitro* infection system, which removes all influences of the immune system, and that may provoke a marked difference in either host or parasite response to an *in vivo* infection. Another plausible explanation is that differences may lie within multigene families which were omitted in this analysis due to the poor current state of assembly of these largely repetitive sequences. It is likely that many of the virulence factors (such as trans-sialidases and MASPs) lie within these multigene families. Using technologies that produce longer sequence reads will lead to a better assembly and annotation, which will resolve differences in multigene families and improve similar analyses in the future. Finally, differences at the proteome level may provide better insights into possible translational regulatory mechanisms not interrogated in this work.

A unique benefit of using RNA-Seq in the context of this work is the ability to characterize the early behavior of both host and parasite simultaneously, even when parasite RNA levels are proportionally very small during the early timepoints of an infection. This study provides a unique look at the host-pathogen interaction between *T*. *cruzi* Sylvio strain and a human host, and the first in-depth comparative transcriptome analysis of two different strains of parasite and their host response.

## Supporting Information

S1 Fig Diagnostic analyses of data- Pearson Correlations. (**A**) Two human samples fell below cutoff of Pearson correlation of 0.8 and were omitted. There is high correlation between all remaining samples. (**B**) The Sylvio samples showed poor correlation with the Y-strain samples, which is likely a biological effect. Samples within strain show high correlation. (**C**). Limiting samples to only the “core” genes (Unique orthologs with reciprocal best hit between Sylvio and Esmeraldo-like TriTrypDB fasta files) greatly improves the correlation.
(TIF)

S1 Table Sample information. This table describes metadata information, total sequenced reads, and mapped reads; these are defined by number and percentage aligned to human, Sylvio, and the Y reference genomes for each sample.
(XLSX)

S2 Table Summary of differential expression analysis for human data. Summary of differentially expressed genes with their direction (up- or downregulated) at various fold change cutoffs.
(XLSX)

S3 Table Differentially expressed human genes. List of all human genes at 24 hpi and 72 hpi along with significance (adjusted for multiple testing) and the fold change for each gene from cells infected with each of the two strains. For each quadrant in Fig 3 which had significantly differentially genes in infections with both strains, and which met the fold change cutoff of >2 (shown in red), additional tabs are provided with a list of those genes. Where GO analysis returned enriched GO categories for those genes, this information is also provided in separate tabs.
(XLSX)

S4 Table Summary of differential expression analysis of parasite data. Summary of differentially expressed core genes with their direction (up- or downregulated) at various fold change cutoffs.
(XLSX)

S5 Table Core parasite genes at 24 hpi compared to 4 hpi. List of all core parasite genes along with significance (adjusted for multiple testing) and the fold change for that gene for each of the two strains.
(XLSX)

S6 Table Core orthologs between *T*. *cruzi* Sylvio strain and Y strain. Final set of orthologs produced for this work. Briefly, Version 8.0 genome fasta files for *T*. *cruzi* Sylvio strain and *T*. *cruzi* CL Brener strain (Esmeraldo-like haplotype) were aligned using the FASTA program ggsearch36, which does a global:global alignment. Reciprocal best hits were then further refined by removing genes in the four largest multi-gene families (MASP, trans-sialidase, retroposon hot-spot and dispersed gene family protein) and then selecting genes which have only a single reciprocal best hit.
(XLSX)
