# Supplementary figures and images for: Comparative Transcriptome Profiling of Human Foreskin Fibroblasts Infected with the Sylvio and Y Strains of Trypanosoma cruzi
Source: PLoS One. 2016 Aug 9;11(8):e0159197. doi: 10.1371/journal.pone.0159197 (PMC4978399; doi:10.1371/journal.pone.0159197)

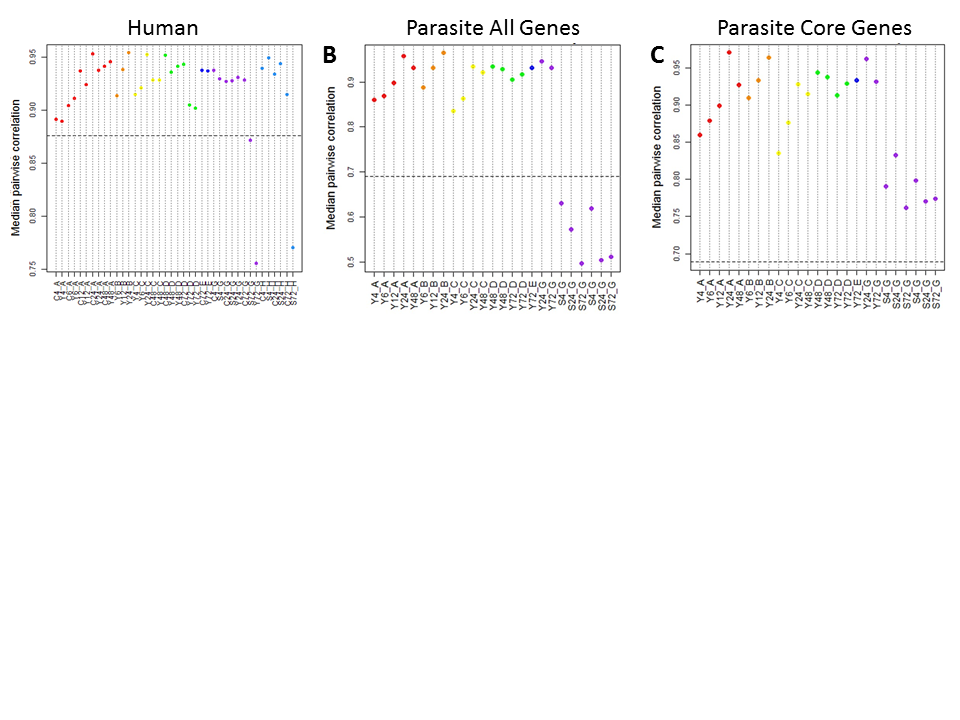

Supplement: S1 Fig — (A) Two human samples fell below cutoff of Pearson correlation of 0.8 and were omitted. There is high correlation between all remaining samples. (B) The Sylvio samples showed poor correlation with the Y-strain samples, which is likely a biological effect. Samples within strain show high correlation. (C). Limiting samples to only the “core” genes (Unique orthologs with reciprocal best hit between Sylvio and Esmeraldo-like TriTrypDB fasta files) greatly improves the correlation. (TIF) [file pone.0159197.s001.tif]
